# Natural Compounds That Target DNA Repair Pathways and Their Therapeutic Potential to Counteract Cancer Cells

**DOI:** 10.3389/fonc.2020.598174

**Published:** 2020-11-19

**Authors:** Francisco Alejandro Lagunas-Rangel, Rosa María Bermúdez-Cruz

**Affiliations:** Department of Genetics and Molecular Biology, Centro de Investigación y de Estudios Avanzados del Instituto Politécnico Nacional (CINVESTAV), Mexico City, Mexico

**Keywords:** DNA damage, radioresistance, chemoresistance, sensitization, treatment

## Abstract

Resistance to current cancer treatments is an important problem that arises through various mechanisms, but one that stands out involves an overexpression of several factors associated with DNA repair. To counteract this type of resistance, different drugs have been developed to affect one or more DNA repair pathways, therefore, to test different compounds of natural origin that have been shown to induce cell death in cancer cells is paramount. Since natural compounds target components of the DNA repair pathways, they have been shown to promote cancer cells to be resensitized to current treatments. For this and other reasons, natural compounds have aroused great curiosity and several research projects are being developed around the world to establish combined treatments between them and radio or chemotherapy. In this work, we summarize the effects of different natural compounds on the DNA repair mechanisms of cancer cells and emphasize their possible application to re-sensitize these cells.

## Introduction

Day by day we are exposed to chemical carcinogens in the environment, ultraviolet (UV) radiation, ionizing radiation, and also those substances produced in our body during cellular metabolism that attack and produce a variety of DNA injuries. Each lesion favors the development of alterations in DNA and chromosomes, which favors oncogenic transformation and tumor progression. In order to reduce the number of changes in the genome and its instability, cells have several pathways of response to damage and DNA repair proteins that eliminate these lesions (1). DNA adducts, such as those created by alkylating agents, can be cleaved and repaired by base excision repair (BER) or by nucleotide excision repair (NER), depending on whether it is necessary to remove only a nitrogenous base or a nucleotide (2). Also, O-6-methylguanine-DNA methyltransferase (MGMT), an alkyltransferase, eliminates alkylations (3). Mismatch repair (MMR) is a system for repairing the insertion, deletion, and misincorporation of bases that can arise during DNA replication and recombination. While, direct double-strand breaks are repaired by non-homologous end joining, those associated with replication are repaired by homologous recombination. Other repair pathways active during replication include the Fanconi anemia repair pathway, endonuclease-mediated repair, and RecQ-mediated repair (2, 4).

**Figure 1:**
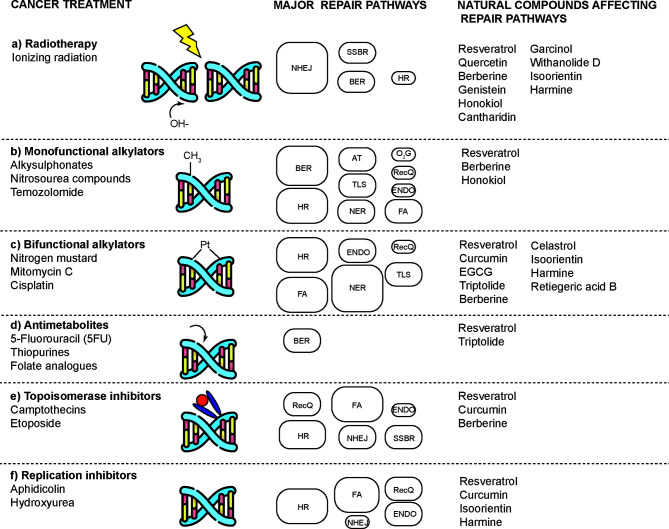
Natural compounds that enhance the effects of radio and chemotherapy by affecting DNA repair mechanisms in cancer cells. DNA damaging agents used in cancer treatment induce a diverse spectrum of toxic lesions. These injuries are recognized by a variety of DNA repair pathways that are specific to the injury but are complementary in some respects. Natural compounds enhance the effects of these toxic agents by preventing proper DNA repair and inducing cell death. DNA repair pathways involved are: base-excision repair (BER), nucleotide-excision repair (NER), alkyltransferases (ATs), mismatch repair (MMR), non-homologous end joining (NHEJ), homologous recombination (HR), endonuclease mediated repair (ENDO), Fanconi anaemia repair (FA), DNA dioxygenases (O2G), and RecQ-mediated repair (RecQ). The size of the boxes represents the relative contribution of each repair mechanism in each type of damage caused by a type of treatment. Modified according to (2).

Several cancer cells in contrast to normal cells have one or more DNA repair pathways defective during carcinogenesis, leading to a greater reliance on the remaining pathways and at the same time accumulating mutations during the process (5). Examples of these are the silencing of MGMT in approximately 40% of glioblastomas (6) and the downregulation of MMR genes in colon cancer (7, 8). However, some types of cancer overexpress DNA repair genes and this makes them more resistant to the treatments currently used, causing what is known as resistance (9). Resistance to current cancer treatments is a major problem that requires the search for new compounds that can re-sensitize cancer cells. We speak of resistance when a cancer cell develops the ability to resist radio and chemotherapy, and this can be achieved through various mechanisms such as regulation of the entry and exit of drugs, inhibition of cell death, alterations in metabolism and degradation of drugs, epigenetic factors, and improved DNA repair (10). In terms of its effects on DNA repair, DNA repair inhibitors have been shown to increase the efficacy of anticancer drugs and several works have illustrated the sensitizing efficacy of natural compounds in various cancers (11). Natural compounds are biologically active substances present in plants, fungi, bacteria, and other organisms that affect DNA repair, and are classified mainly according to their chemical structure into terpenes, carotenoids, phenolic compounds (**Table 1**): phenolic acids, flavonoids, stilbenes, coumarins, tannins; alkaloids, nitrogen compounds; organosulfates: isothiocyanates and indoles, allyl sulfates. Flavonoids are further divided into chalcones, flavanones, flavones, flavonols, flavanols, isoflavones, and anthocyanins (12). In this work, we summarize the effects of different natural compounds on the DNA repair mechanisms of cancer cells and emphasize their possible application to re-sensitize these cells to radio and chemotherapy (**Figure 1**).

**Table 1:** Structural classification of natural compounds targeting DNA repair pathways in cancer cells.

Class	Active metabolite	Structure
Phenolic compounds	Curcumin	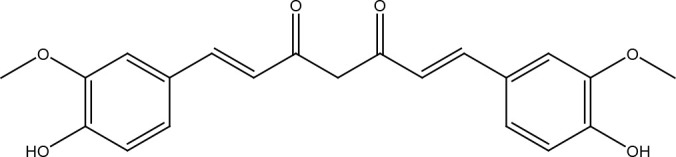
	Epigallocatechin gallate (EGCG)	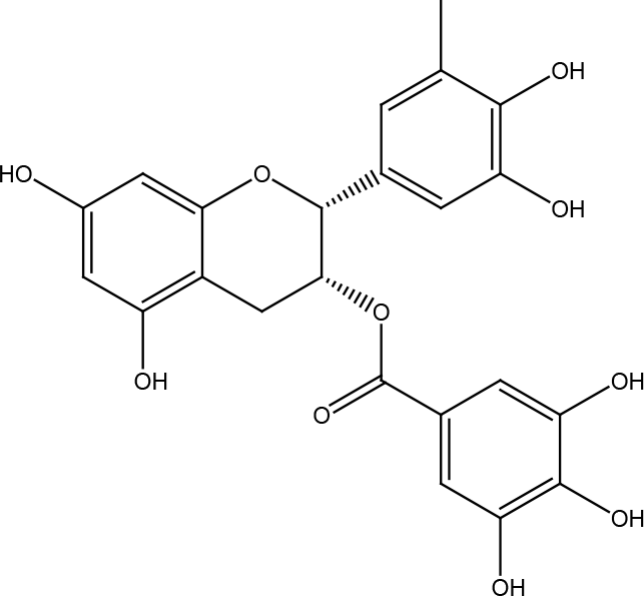
	Genistein	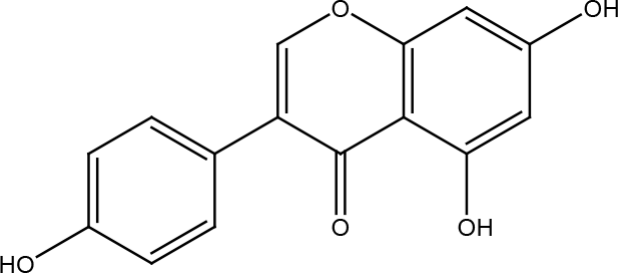
	Quercetin	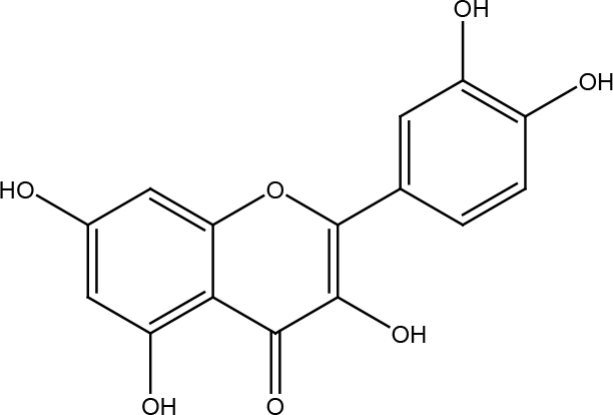
	Resveratrol	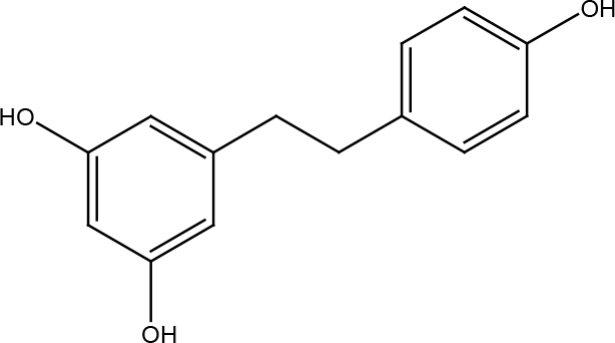
	Honokiol	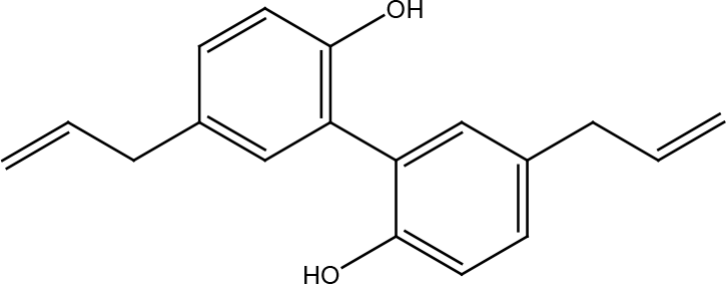
	Ellagic acid	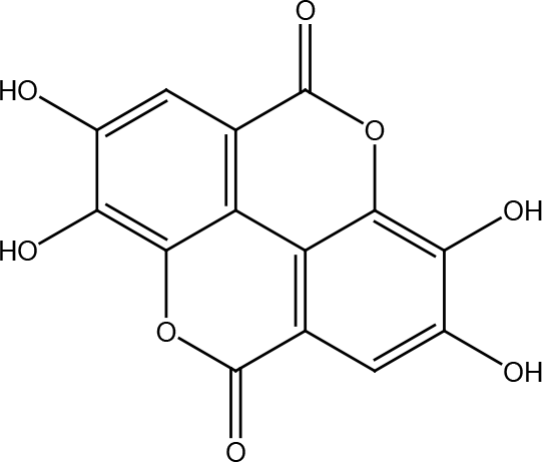
	Kaempferol	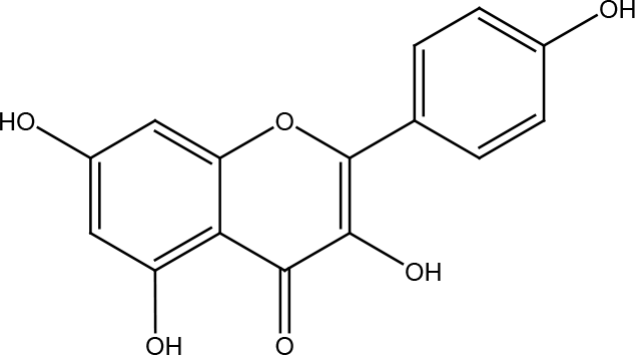
	Isoorientin	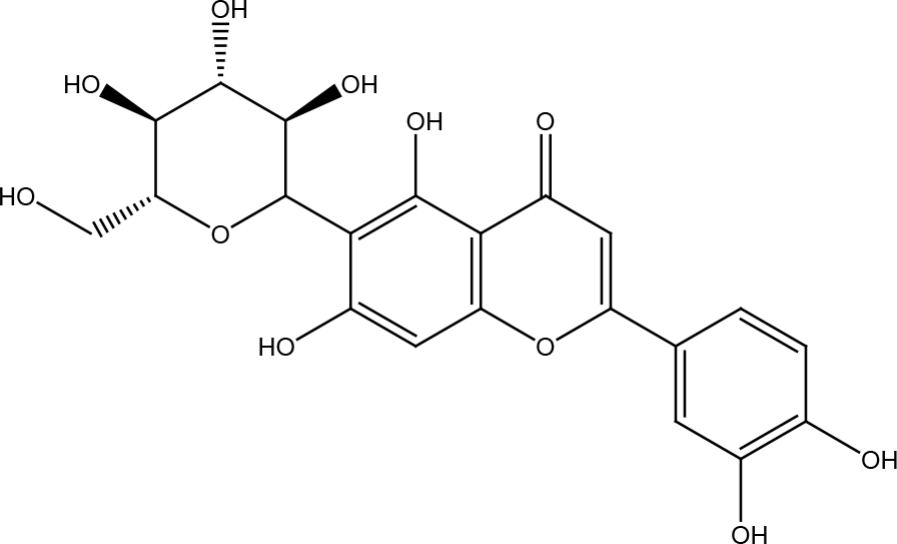
	Ferrulic acid	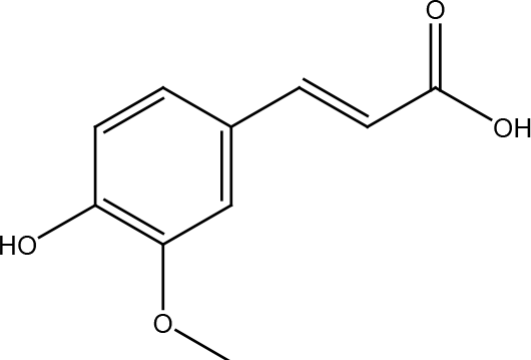
Terpenoids	Celastrol	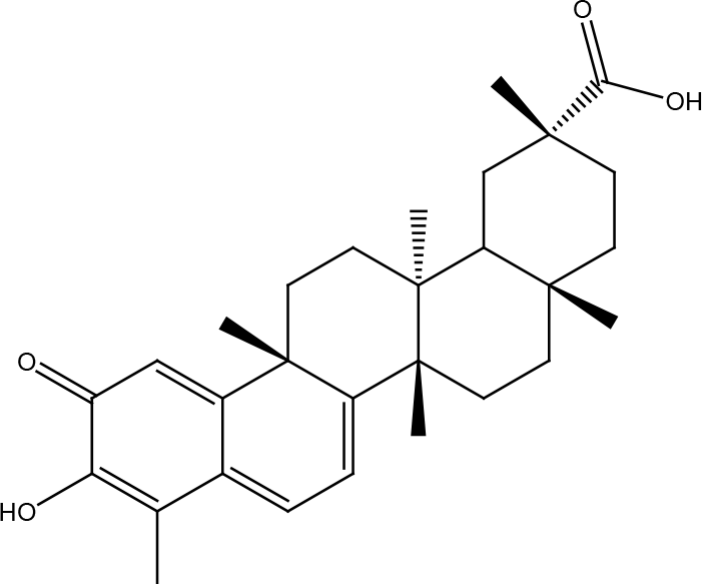
	β-Carotene	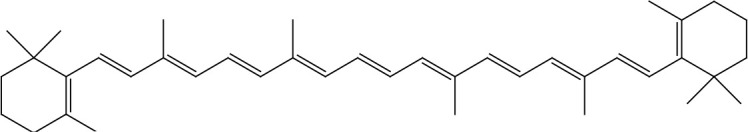
	Triptolide	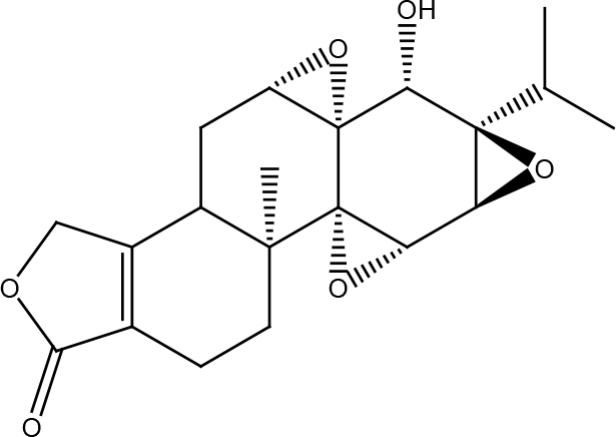
	Cantharidin	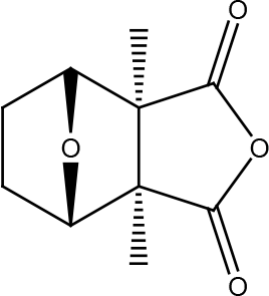
	β-Thujaplicin	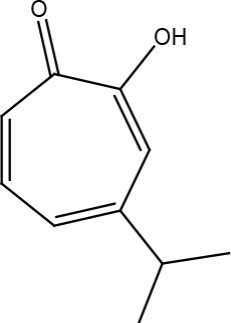
	Retigeric acid B	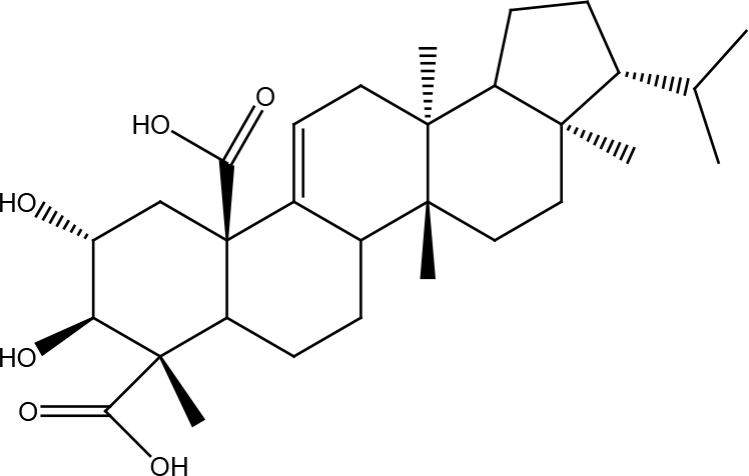
	Thymoquinone	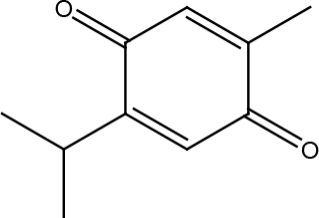
	Withanolide D	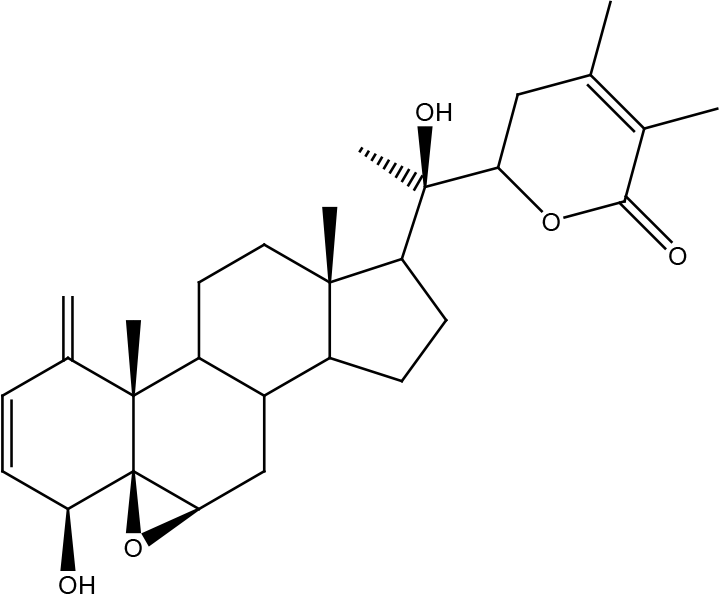
	Garcinol	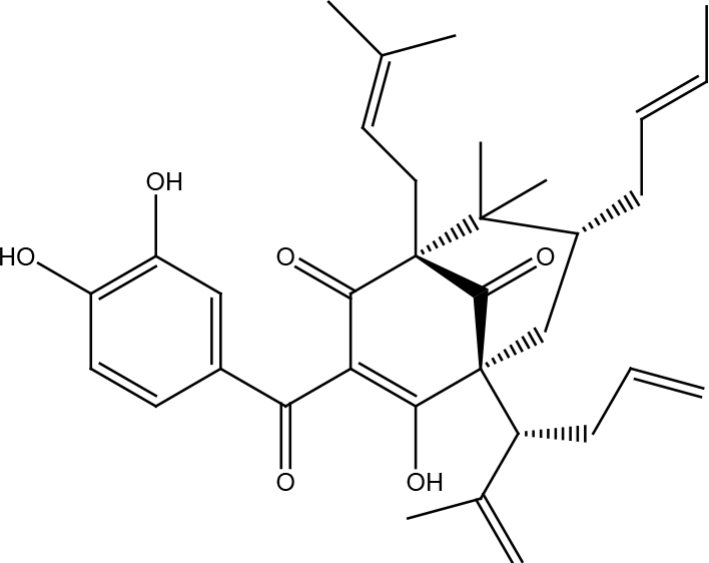
Nitrogen-containing alkaloids	Berberine	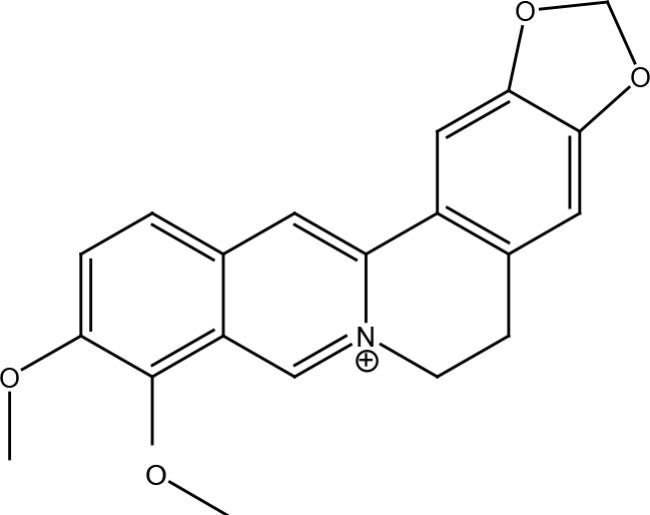
	Capsaicin	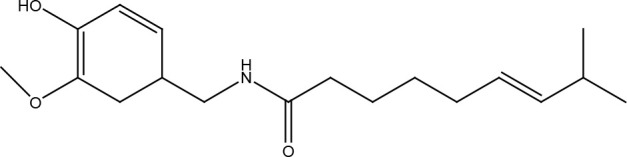
	Harmine	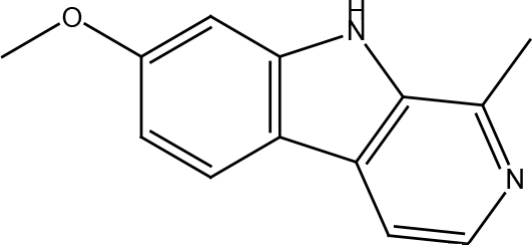

## Resveratrol

Resveratrol is a natural polyphenolic compound, specifically a stilbene, which is found in significant amounts in grapes, berries, peanuts, and other plant sources, as well as in red wine. This compound has become very popular due to its multiple reported properties that include inflammation-mediating, cardioprotective, antioxidant, and anti-cancer, among other things (13). As an anti-cancer compound, low-dose resveratrol accelerates non-mutagenic repair of DNA damage in mouse embryonic stem cells exposed to ionizing radiation (14). Similarly, resveratrol in mouse embryonic fibroblasts was shown to help maintain genomic stability after chemical and ionizing radiation damage by allowing greater repair efficiency of double-strand breaks and less replicative stress (15). Furthermore, resveratrol was shown to significantly reduce DNA damage from arsenic compounds in non-cancerous mammalian cells by enhancing repair activities, especially if used prior to exposure (16). Resveratrol causes DNA damage and activates the repair mechanisms in various cancer cell lines such as prostate cancer cells, colon cancer cells, and breast cancer cells (17, 18). Indeed, head and neck squamous cell carcinoma cells as well as breast cancer cells receive more DNA damage than their normal counterparts (19, 20). Non-small cell lung cancer cells have shown DNA damage after treatment with resveratrol, which was potentiated by the pemetrexed antifolate that destabilizes ERCC1 protein, an essential nuclease in the BER pathway and, to a lesser extent, in double-stranded DNA breaks and in crosslink repair, by inhibiting p38 MAPK activity (21). Resveratrol has been shown to affect different DNA repair pathways in MCF7 breast cancer cells by reducing the expression of several genes involved in this activity and where mismatch repair and homologous recombination stand out such as most affected (22). Resveratrol made breast cancer cells more susceptible to cisplatin, and specifically in cisplatin-resistant MCF7 cells, resveratrol was able to re-sensitize cells by decreasing several key components of the homologous recombination pathway (23). Etoposide in combination with resveratrol treatments were more effective than either chemical alone given as treatment to stop cell proliferation and eliminate non-small-cell lung cancer cells by suppressing the expression of the XRCC1 protein (DNA repair protein within NER or BER pathway) (24). The same happened in sphere cultures of cervical cancer cells treated with this combination, but in this case a strong decrease in the expression of the RAD51 protein (DNA repair protein within HR pathway) was reported (25). Resveratrol potentiates the effects of temozolomide on glioblastoma cells by negatively regulating the NF-κB pathway and thereby causing a reduction in MGMT expression (26). Resveratrol switched radioresistant prostate cancer cells back to sensitive phenotype by inhibiting ATM phosphorylation and its target protein H2AX, causing cell cycle arrest and subsequently cell death (27). Resveratrol also radiosensitized glioma stem cells by causing an accumulation of DNA damage that impairs their self-renewal and potency (28). By the same mechanism, resveratrol together with capsaicin made radiosensitive pancreatic tumor cells more susceptible to the effect of radiation (29). In colon cancer cells resistant to 5-fluorouracil, resveratrol in conjunction with 1,3-bis(2-chloroethyl)-1-nitrosourea (BCNU) managed to induce apoptosis and re-sensitize the cells by decreasing the levels of FEN1 and PCNA (30). The same decrease in both proteins was observed in cigarette smoke-induced breast cancer cells treated with resveratrol alone, where it was also detailed that p21 levels increased and affected the binding of FEN1 to PCNA, thus inhibiting the long patch base excision repair pathway. Other components of this pathway, such as DNA-ligase-I and polymerases (β, δ, ϵ) were also decreased (31). Despite the fact that melanoma cells have an increased expression of APE/REF1, especially those resistant to dacarbazine, it has been shown that resveratrol can sensitize them by inhibiting REF1-activated AP-1 DNA bindings (32). As can be seen from the data referred, resveratrol is an important candidate despite somewhat solubility issues that affect its bioavailability.

## Curcumin

Curcumin is a bright yellow hydrophobic polyphenol present in the rhizome of turmeric (*Curcuma longa*) and to which antimicrobial, anti-inflammatory, antioxidant, immunomodulatory, renoprotective, hepatoprotective, hypoglycemic, and anti-cancer effects have been attributed (33). Curcumin’s ability to affect multiple pathways makes it an extremely powerful anticancer agent. Furthermore, curcumin has shown multiple effects on DNA repair systems, both in healthy cells and cancer cells. Curcumin prevents DNA damage in lymphocytes of people chronically exposed to arsenic and improves its repair capacity. Thus, it induces an increase in the proteins of the base excision repair and non-homologous end joining pathways and collaborates to avoid carcinogenesis (34). Also, in murine models, curcumin reduced cyclobutane and pyrimidine dimers produced after exposure to UVB radiation and delayed skin carcinogenesis (35). In cancer cells, curcumin blocks both non-homologous end joining and homologous recombination pathways: by inhibiting the acetyltransferase activity of CBP on histone at double strand breaks thus preventing the recruitment of KU70/KU80 proteins and p300 on BRCA1 promoter and causing downregulation of its expression. ATR kinase activity is also inhibited by curcumin, causing cell cycle arrest in the G2 phase (36, 37). It has also been seen that mismatch repair is important in curcumin activity because cells deficient in this system, particularly when MSH2 and MLH1 proteins are affected, show a greater sensitivity to it. The difference is that the competent cells of the mismatch repair system can activate CHK1 and arrest in the G2/M phase before inducing apoptosis, whereas the deficient cells go directly to apoptosis (38). In gastric cancer cells, curcumin induces DNA damage that is reflected by overexpression of DNA-PKcs, ATM, ATR, HDAC1, p21, and GADD45A along with activation of the p53 pathway, which consequently suppresses phosphorylation of Rb and expression of cyclin E, thus stopping the cell cycle and causing a general demethylation of DNA by repressing the expression of DNMT1 thus allowing the re-expression of tumor suppressor genes (39). The same effect on DNMT1 was reported in curcumin-treated breast cancer cells, but the effects were different between cell lines. For example, in HCC-38 cells, the curcumin-dependent decrease in DNMT1 together with the inhibition of miR-29b caused an increase in TET1 (a methylcytosine dioxygenase that plays an important role in the demethylation of DNA) allowing BRCA1 re-expression, but this did not occur in T47D cells (40). It is also important to note that the response to DNA damage triggered by curcumin and varies according to the BRCA1 mutation status in triple negative breast cancer cells, but regardless of this, in all cases it leads to apoptosis (41). In curcumin-treated MCF-7 breast cancer cells, a decrease in FEN1 (long patch BER pathway) was observed as a result of overexpression of NRF2 and its positioning on the promoter of this gene, thus collaborating to prevent cell proliferation (42). In lung cancer cells, curcumin reduces the levels of some DNA repair proteins such as BRCA1, MGMT, MDC1, and 14-3-3σ, but elevates DNA damage proteins such as phosphorylated p53 and γH2AX, thus causing cytotoxicity, condensation of the nucleus, and DNA damage (43). Meanwhile, curcumin causes DNA damage in cervical cancer cells and increases levels of BRCA1, MGMT, MDC1, p53, DNA-PKcs, MDM2, PARP, and the phosphorylated forms of ATM, ATR, and H2AX (44). In contrast, RAD51 foci formation was also decreased in lymphoma cells and breast cancer cells treated with curcumin (45, 46).

On the other hand, the ability of curcumin to reverse chemoresistance in various cancers is remarkable. In combination with cisplatin, curcumin prevents the activation of p38 MAPK through MKP1 phosphatase activity consequently affecting the expression of XRCC1, making lung cancer cells more sensitive to the cytotoxic effects of this chemotherapeutic agent (47). A decrease in thymidine phosphorylase, ERCC1 and RAD51 can also be observed with this combination and with mitomycin C and curcumin, which is due to the inhibition of ERK1/2 activity and an increase in their ubiquitin-mediated 26S proteasome degradation (48, 49). As a complementary medicine to carboplatin, curcumin reduces its adverse effects by selectively activating nucleotide excision repair and homologous recombination in bone marrow cells through positive regulation of BRCA1, BRCA2, and ERCC1 expression, but it has the opposite effect on malignant cells (50). Together with quinacrine, curcumin binds DNA more efficiently, being able to cause further damage to breast cancer stem cells and preventing their repair by lowering the expression of DDB2, Polβ, Polδ, PolH, Rad51, Fen1, XRCC1, CHK1, and RPA proteins (51). Curcumin increases the apoptotic effects of cisplatin on cisplatin-resistant lung adenocarcinoma cells by inhibiting FANCD2 monoubiquitination and, therefore, also preventing activation of the Fanconi anemia/BRCA pathway that enables DNA repair by homologous recombination (52). The same effect was reported in multiple myeloma cells treated with melphalan and curcumin (53). Curcumin sensitizes colon cancer cells to radiation by modifying the expression of several genes, highlighting an overexpression of CCNH and XRCC5 along with low expression of LIG4 and PNKP (54). Hydroxyurea, camptothecin, and cisplatin were shown to be more efficient in lymphoma cells when combined with curcumin (45). In the same way, PARP inhibitors and DNA-PK inhibitors together with curcumin showed a synergistic effect to induce DNA damage, apoptosis, and mitotic cell catastrophe in different cancer cell lines (36, 45, 46). This, in part, due to the inhibition of topoisomerase II and the reduction in the expression of WRN, FEN1, APE1, DNA ligase III, and XRCC1 (55).

## (—)-Epigallocatechin-3-Gallate

The main polyphenolic component of green tea (*Camellia sinensis*) extracts is epigallocatechin gallate (EGCG), an ester of epigallocatechin and gallic acid, and a type of catechin. Biological effects that have been reported for EGCG are antioxidant, anti-inflammatory, neuroprotective, cardioprotective, and anti-cancer (56). In terms of anti-cancer effects, among the many activities that EGCG has (57), some of them are related to its effect on DNA repair systems. EGCG is a compound capable of inhibiting the activity of the ERCC1/XPF protein in non-small cell lung cancer cell lines, blocking the intrastrand crosslink repair, and thus enhancing the cytotoxic activities of cisplatin, preventing proliferation and increasing cellular death (58). Furthermore, EGCG selectively decreased MGMT levels in glioblastoma multiforme cells by preventing translocation of β-catenin to the nucleus, thereby avoiding the removal of temozolomide-produced O6-methylguanine and helping to resensitize cells resistant to this drug. In contrast, EGCG improved MGMT expression in non-tumor glial cells by inhibiting DNMT1 and allowing demethylation of its promoter (59). Normal human leucocytes with continuous low-dose EGCG treatments show less DNA damage (single and double chain mutations, adducts, and mutations) when exposed to genotoxic agents such as bleomycin and some heterocyclic amines (60, 61).

## Triptolide

Triptolide is a diterpene triepoxide obtained from the Chinese medicinal plant *Tripterygium wilfordii* Hook F, commonly known as lei gong teng or thunder god vine. This compound has a variety of bioactivities and pharmacological effects such as anti-microbial, anti-inflammatory, neuroprotective, cardiovascular, immunosuppressive, and recently anti-cancer (62). The anticancer effects of triptolide are time and dose dependent, varying according to cell type, but where its effects on DNA repair mechanisms stand out, most often culminating in apoptosis of cells. First, triptolide was shown to affect the nucleotide excision repair pathway by selectively inhibiting the ATPase activity of XPB helicase, thus allowing for a malfunction of the TFIIH holocomplex and preventing filling of the gaps after damage excision (63). Then, triptolide was reported to inhibit the double-stranded DNA damage response in breast cancer cells through post-transcriptional downregulation of ATM, which causes a reduction in the levels of γH2AX (64). The same was observed in melanoma cell lines along with decreased levels of ATR, BRCA-1, DNA-PKcs, MGMT, and p53 (65). Meanwhile, in murine B−cell lymphoma cells and acute lymphoblastic leukemia cells, triptolide induces DNA double strand breaks with upregulation of γH2AX and RAD51, which culminates in caspase-3 dependent apoptosis and helps enhance the effects of PARP1 and PI3K inhibitors, as well as re-sensitizing cytarabine- and doxorubicin-resistant leukemia cells (66, 67). Triptolide was shown to cause a decrease in the levels of PARP1, XRCC1, and RAD51 proteins in triple negative breast cancer cells, affecting single-strand break repair, base excision repair, and homologous recombination pathways (64). Furthermore, this natural compound causes cells accumulate DNA damage, stopping their growth, and being arrested in the S phase of the cell cycle, as well as presenting a greater sensitivity to chemotherapeutic agents such as cisplatin and doxorubicin (64, 68). Lung cancer cells showed an increase in ATM phosphorylation after combined treatment of cisplatin with triptolide, which led to the activation of apoptotic genes such as PUMA (69). Likewise, triptolide showed synergy with oxaliplatin in pancreatic cancer cell lines by producing a decrease in the expression of key proteins in the nucleotide excision repair pathway such as XPA, XPB, XPC, ERCC1, XPD, and XPF, but unlike breast cancer cells, here showing an increase in the levels of γH2AX and, therefore, also of DNA double strand breaks (70).

## Quercetin

Quercetin is a flavonoid found in a variety of foods, including fruits and vegetables such as apples, berries, capers, grapes, onions, shallots, tea, and tomatoes, as well as many seeds such as nuts, flowers, bark, and leaves (71). Quercetin is known for its anti-inflammatory, antihypertensive, vasodilatory, anti-hypercholesterolemic, anti-atherosclerotic, antioxidant and, more recently, anti-cancer effects (72). Quercetin following a 1,2-dimethylhydrazine dihydrochloride (DMD) induced colon carcinogenesis protocol allowed decreased production of 8-oxoguanine and apurine/pyrimidine sites by increasing levels of the BER proteins OGG1, APE1, and XRCC1, and positively modulate NRF2 signaling with a higher antioxidant response (73). Also in response to oxidative damage to colon cells by H_2_O_2_, an increase in OGG1 was observed (74). In prostate cancer cells, quercetin significantly reduced the expression of ATM, PARP1, and DNA-PKcs (75). Quercetin suppresses the repair of double-stranded DNA breaks and improves the radiosensitivity of ovarian cancer cells through activation of ATM and the p53-dependent endoplasmic reticulum stress pathway (76). Meanwhile, in some colorectal cancer, cervical cancer and breast cancer cell lines, quercetin acted as a radiosensitizer by blocking ATM activation and its downstream signaling, thereby prolonging the persistence of damage and inducing apoptosis (77). Quercetin can potentiate the effects of PARP inhibitors, preventing efficient repair of DNA damage, and where inhibition of BRCA2 activity plays an important role during the passage of single-strand breaks to double-strand breaks during DNA replication (78).

## Berberine

Berberine is an isoquinoline alkaloid isolated mainly from the Chinese herb *Coptis chinensis*, although it is also present in other plants of the genus *Berberis*. It has a wide range of pharmacological properties such as anti-inflammatory, antibiotic, antitumor, antiarrhythmic functions, and it can regulate blood lipids and glucose levels (79). Berberine has been shown to induce oxidative DNA damage and alter RAD51 expression in ovarian cancer cells, breast cancer cells, and osteosarcoma cells, but not in normal cells, thereby causing increased DNA damage and longer, with abundant γH2AX, ATM, and p53 foci (80–82). This property has been important in radiosensitizing breast cancer cells and esophageal cancer cells (82, 83). Furthermore, it showed synergy with PARP inhibitors to induce cellular apoptosis (80). Also, berberine was able to increase the sensitivity of triple negative breast cancer cells to cisplatin, camptothecin, and methyl methanesulfonate by attenuating XRCC1-mediated repair of base excision and subsequently increasing double-stranded DNA breaks (84).

## Genistein

Genistein is a multifunctional isoflavonoid whose best-known source is soy-based foods. Genistein has been shown to modulate various pathways involved with obesity, metabolic syndrome, and cancer (85). In normal skin, genistein reduces the formation of cyclobutane pyrimidine dimers caused by UVB radiation (86), and in rats treated with genistein, BRCA1 expression was elevated and tumorigenesis caused by 7,12-dimethylbenz [a] anthracene (DMBA) was reduced (87). Genistein inhibited both homologous recombination repair and non-homologous end joining pathway in glioblastoma cells and sarcoma cells after the damage caused by the radiation of carbon ions. This can be explained by considering that genistein prevents the phosphorylation of DNA-PKcs and KU80, and it delays the formation of RAD51 foci (88, 89). The same happened with X-ray therapy and a combined treatment of genistein and IGF1R inhibitor AG1024 in prostate cancer cells (90). Genistein has also been shown to reduce AP-1 levels and sensitize these cells to doxorubicin nanoparticles (91). Interestingly, normal liver cells were protected from damage by ionizing radiation using low concentrations of genistein (92).

## Other Compounds

Thymoquinone is the main active component of *Nigella sativa Linn* seed extracts and has been shown to possess antineoplastic properties. This compound induces DNA damage and apoptosis in glioblastoma cells where shortening of telomeres is involved by a DNA-PKcs-dependent mechanism (93). Honokiol is a biphenolic compound with a powerful antineoplastic activity which is obtained from the *Magnolia officinalis* plant. It is more toxic in tumor cells than in normal cells and has been reported to inhibit the activity of the X family polymerases (β and λ), affecting the base excision repair pathway and making various cancer cells more susceptible to the effect of bleomycin and temozolomide (94). Ellagic acid obtained from various fruits and vegetables is a polyphenolic compound that can reduce MGMT expression in glioblastoma cells and together with anti-angiogenic therapy with bevacizumab (which also affects DNA repair by reducing the expression of ERCC-1 and XRCC-1) improves the radiosensitivity of tumors (95, 96). Celastrol is a polyphenolic compound isolated mainly from plants in the *Celastraceae* family. Celastrol has been shown to exhibit significant antioxidant, anti-inflammatory, and antineoplastic activities. For this last aspect, celastrol promotes a reduction in cancer cells of the monoubiquitinated FANCD2 protein, promoting its degradation by the proteasome and affecting the activation of the DNA damage-induced Fanconi anemia pathway and the downstream pathways. Thus, enhancing the effects of crosslinking agents such as cisplatin (97). Cantharidin is a substance of the terpenoid class that is secreted by many species of blister beetles, and which has been observed to sensitize pancreatic cancer cells to the effects of ionizing radiation by increasing levels of phosphorylated H2AX and affecting the expression of UBE2T, RPA1, GTF2H5, LIG1, POLD3, RMI2, XRCC1, PRKDC, FANCI, FAAP100, RAD50, RAD51D, RAD51B, and DMC1, which are important for repair by homologous recombination and non-homologous end joining pathway (98). In bladder cancer cells, decreased phosphorylated ATR and H2AX, as well as total levels of DNA-PK, PARP, MGMT, BRAC1, and MDC1 were observed with this compound (99). Garcinol, a polyisoprenylated benzophenone derivative of the fruit rind *Garcinia indica*, sensitizes cervical cancer cells to ionizing radiation by inhibiting non-homologous end joining pathway by preventing chromatin remodeling, especially histone acetylation (100, 101). Gastric cancer cells treated with high doses of β-carotene showed a significant decrease in the KU70 and KU80 proteins (102). Androgen receptor-target DNA repair genes were epigenetically repressed in androgen-sensible prostate cells after treatment with 3,3’-diindolylmethane, a compound derived from indole-3-carbinol and found in cruciferous vegetables such as broccoli, brussels sprouts, cabbage, and kale (103). Kaempferol inhibited the expression of DNA-PKcs, MDC1, MGMT, p53, 14-3-3, phosphorylated forms of ATM and ATR in promyelocytic leukemia cells but increased phosphorylated p53 and H2AX. Kaempferol is a flavonoid found in vegetables and fruits such as berries, grapefruit, and *Ginkgo biloba* (104). Luteolin, a flavonoid enriched in various vegetables and plants such as carrots, broccoli, and parsley, reduced phosphorylation levels of ATM, CHK2, and H2AX in oral squamous cell carcinoma cells (105). In lung squamous carcinoma cells, luteolin caused an increase in the levels of MHT1, OGG1, and AP-1 (106). Withanolide D, a compound obtained from *Withania somnifera*, was shown to improve the radiosensitivity of different cancer cell lines by inhibiting DNA damage *via* non-homologous end joining repair pathway (107). Isoorientin is a flavonoid extracted from many plant species, such as flax straw, watery leaf, *Gypsophila elegans*, *Phyllostachys pubescens*, *Patrinia*, and *Drosophyllum lusitanicum*. Meanwhile, harmine is a tricyclic β-carboline alkaloid that was originally isolated from *Peganum harmala* seeds. Both compounds inhibited repair by homologous recombination in hepatoma cells, without affecting normal cells, by inhibiting the ATM-downstream signaling pathways and therefore enhancing the effects of ionizing radiation, hydroxyurea, mitomycin C, olaparib, and camptothecin (108, 109). Ferulic acid potentiated the effects of PARP inhibitors on breast cancer cells by reducing the formation of RAD51 foci and lengthening the time that double-stranded DNA breaks remain unrepaired (110). Capsaicin, the main bioactive compound found in chili peppers of the *Capsicum* genus, downregulates the ERCC1 protein in lung cancer cells by promoting its proteasomal degradation, thereby enhancing the cytotoxic effects of the EGFR inhibitor erlotinib (111, 112). β-Thujaplicin, a natural monoterpenoid found in the wood of trees in the *Cupressaceae* family, sensitized osteosarcoma cells to damage caused by ionizing radiation, as it inhibits the formation of RAD51 foci and keeps RPA phosphorylated (113). Retiegeric acid B potentiates the effects of cisplatin on hormone-refractory prostate cancer cells by affecting nucleotide excision repair, particularly ERCC1, TFB5, and RPA1 proteins, and mismatch repair, presumably MSH2 and MSH6 proteins (114).

## Conclusions

Natural compounds have been be used with other drugs to make cancer cells more sensitive to radiation therapy and different chemotherapeutic agents, even reversing the resistance mechanisms that these cells may have developed. Since increasing the levels of genes involved in DNA repair is a mechanism used by many cancer cells to resist the effects of radio and chemotherapy, the fact that natural compounds can affect the DNA repair pathways makes them candidates to reverse cases of resistance and thus, perhaps contribute to the improvement of patients to allow their survival time to be longer. Despite this potential, there are currently very few clinical trials testing these compounds in combination with chemotherapy or radiotherapy, mainly due to all the challenges that this entails [revised in (115)], including shortages of funds due to lack of patentability and manufacturing difficulties. It is necessary to continue studying different natural compounds and their effects on DNA repair systems in order to implement them in current treatment strategies, establish the appropriate doses and times, and decipher the mechanisms of action by which they carry out their effects.

## Author Contributions

FL-R compiled the information, wrote the manuscript, made the figures and tables, RB-C reviewed and corrected the manuscript as well as obtained funding. All authors contributed to the article and approved the submitted version.

## Conflict of Interest

The authors declare that the research was conducted in the absence of any commercial or financial relationships that could be construed as a potential conflict of interest.
